# Effect of Multiradiance Low-Level Laser Therapy and Topical Silver Sulfadiazine on Healing Characteristics of Dermal Wounds in Marine Toads (*Rhinella marina*)

**DOI:** 10.1155/2020/8888328

**Published:** 2020-10-17

**Authors:** Kate E. Archibald, Tara Harrison, Brigid Troan, Dustin Smith, Larry J. Minter

**Affiliations:** ^1^Department of Clinical Sciences, College of Veterinary Medicine, North Carolina State University, 1060 William Moore Drive, Raleigh, NC 27606, USA; ^2^Hanes Veterinary Medical Center, North Carolina Zoological Park, 4401 Zoo Parkway, Asheboro, NC 27205, USA; ^3^Environmental Medicine Consortium, North Carolina State University, College of Veterinary Medicine, 1060 William Moore Drive, Raleigh, NC 27607, USA; ^4^The Maryland Zoo in Baltimore, 1876 Mansion House Drive, Baltimore, MD 21217, USA

## Abstract

Current recommendations for wound management in amphibians are based primarily on clinical experience and on extrapolation from other taxa, whereas controlled clinical studies are lacking. Low-level laser therapy, also termed photobiomodulation, has gained popularity in veterinary medicine and may represent a valuable adjunct therapy for wound care in amphibians, though dosing and safety evaluations have not been previously reported. Silver sulfadiazine (SSD), a topical antimicrobial, is commonly utilized in amphibian medicine but little is known about its effects on wound healing in this class of animals. This pilot study evaluated the effects of repeated treatments of low-level laser therapy or topical SSD on second-intention healing characteristics of surgically induced full-thickness dermal wounds in 33 adult wild-caught marine toads. Toads were anesthetized, and a 6 mm cutaneous biopsy was performed over the right dorsum. They were then randomly assigned to one of three groups: laser therapy (LT) at 5 Hz (905 nm wavelength on a super pulsed sequence), topical SSD (SD), or control sham treatment (CT). Treatments were administered at 24 hrs after biopsy and then every 72 hrs thereafter, concurrent with a visual assessment of the wound. Toads were euthanized at one of five timepoints (day 4, 7, 13, 19, or 28) to permit scoring of histologic criteria, including lymphocytic inflammation, granulomatous inflammation, heterophilic inflammation, granulation tissue, fibrosis, and reepithelialization. Visual assessments and histologic scoring did not identify a benefit of laser therapy or SSD as compared to controls. Laser therapy and SSD, at the doses and dosing schedule utilized in this pilot study, appear to be safe and well-tolerated treatments in marine toads, but may not be warranted for uncomplicated skin wounds in this species.

## 1. Introduction

Dermal injuries occur commonly in captive amphibians due to their relatively thin skin and lack of protective dermal structures such as thickened keratin, hair, or scales. Basic wound management principles are often applied to amphibians; however, wound care presents unique challenges due to their dermal physiology. Amphibian skin is a highly specialized organ that contributes to homeostatic functions including water balance, electrolyte exchange, and respiration [1, 2]. As a result, challenges encountered in amphibian wound management include the systemic absorption of topical medications, the disruption of osmoregulation, and direct contact of wound beds with aquatic environments [3, 4]. Furthermore, it is difficult to employ the current standards of wound care in veterinary medicine (provision of an aseptic, moist wound environment) due to the physical impracticality of applying bandages to most amphibian species [5].

Studies evaluating wound healing in amphibians have focused primarily on the mechanisms of limb regeneration, limited scar formation, and the antimicrobial qualities of amphibian dermal secretions [6–14], rather than specific treatment modalities. Published recommendations for wound management in amphibians have been based on clinical experience and on extrapolation from other taxa [3, 15–17], whereas controlled clinical studies evaluating these management strategies are lacking.

Silver sulfadiazine (SSD) is a broad-spectrum topical antimicrobial agent commonly utilized in herpetological medicine. It is active against many Gram-positive and Gram-negative bacteria, namely, *Pseudomonas* spp., as well as some fungal organisms [18]. It is an attractive medication for topical wound management in amphibians not only for its spectrum of activity, but also because it comes with a water-miscible base making it less likely to disrupt amphibian respiratory and osmoregulatory functions as compared to petroleum-based products [15–17]. Due to its widespread use, critical evaluation of the utility of SSD for wound management in this class of animals is warranted.

More recently, the use of low-level laser therapy (LLLT) has become widespread in veterinary practice, including use in nondomestic species [19–22]. Laser therapy, also termed photobiomodulation, is the application of near-infrared laser light on tissue to impart therapeutic benefits. Though not yet fully characterized, the biological effects are reported to involve the enhancement of cellular growth, cellular differentiation, prostaglandin synthesis, acceleration of the inflammatory response, increased rate of granulation tissue contraction, and increased production of proteins such as collagen that contribute to wound healing [23, 24]. LLLT is proposed to enhance the treatment of surgical incisions, open wounds, musculoskeletal injuries, and postoperative pain [25–27]. Despite overall inconsistent methodologies in numerous *in vitro* and *in vivo* studies evaluating laser therapy, findings indicate that LLLT can improve healing time in mammals [28–30]. Specifically, LLLT accelerated wound healing in rats, mice, pigs, horses, dogs, and humans [29, 31–35]. Given the complexities of wound care in captive amphibians, LLLT may represent a valuable noninvasive wound management tool. However, amphibian-specific dose recommendations and safety evaluations have not yet been published.

The purpose of this pilot study was to determine the effect of repeated treatments of LLLT and topical SSD on the second-intention healing characteristics of surgically induced full-thickness dermal wounds in wild-caught marine toads. A secondary objective was to evaluate the tolerability and safety of LLLT application in this species.

## 2. Materials and Methods

### 2.1. Animals

Thirty-four (female *n* = 6, male *n* = 28) adult marine toads were collected from a free-ranging invasive population found at a zoological institution in Miami, Florida. All toads were captured as part of a routine population management program and then transferred to the North Carolina Zoo for inclusion in this study. Animals were identified via a Passive Integrated Transponder (PIT) tag (Biomark Inc., Boise, ID 83702, USA) placed subcutaneously in the rear limb.

For inclusion in the study, animals had to be ≥90 g and appear outwardly healthy. Housing and husbandry were provided according to the established protocols for amphibians quarantined at the North Carolina Zoo. The toads were housed indoors divided into three 135-gallon tanks (70” × 32” × 14”) each provided with a hide area and pool containing reconstituted reverse osmosis (RO) water. The tanks were cleaned daily and disinfected once weekly. In addition to artificial fluorescent lighting, a focal UV basking light was placed over each tank. The light cycle was maintained according to the working hours of the animal husbandry staff (8:00 AM–5:00 PM). The area housing the study animals was not climate-controlled and indoor temperatures fluctuated daily and throughout the 28-day study period (approximately 6.7–30.6°C, 44–87°F) in accordance with the outdoor temperatures (1.7–30.6°C, 35–87°F). The toads were fed gut-loaded crickets dusted with calcium powder three times weekly and were supplemented with commercial cat food (Cat Chow Complete, Nestle Purina PetCare Co., St. Louis, MO 63102, USA) two to three times per week. Prior to the initiation of the study, a random subset of five animals were tested for the presence of *Batrachochytrium dendrobatidis* DNA via PCR of dermal swabs and were found to be negative. Routine fecal analysis prior to the study revealed rare-to-moderate numbers of *Strongyloides* spp. ova, as would be expected for wild-caught animals. Animals were visually examined daily and weighed once weekly throughout the study. The toads were given a five-week acclimation period prior to the initiation of the study. Animals that did not gain weight during that period (*n* = 6) were treated empirically with a single dose of ivermectin (ProMectin, Vedco Inc., Saint Joseph, MO 64507, USA) 0.2 mg/kg IM.

This study was approved by Zoo Miami's Research Committee, North Carolina Zoo's Research Committee, and the North Carolina State University's Institutional Animal Care and Use Committees (IACUC ID 17-119-O).

### 2.2. Anesthesia and Surgery

On day 0, toads were individually placed under general anesthesia to facilitate wound formation via surgical biopsy. Anesthesia was induced via partial immersion in tricaine methanesulfonate (MS-222) (Tricaine-S, Western Chemical Inc., Ferndale, WA 98248, USA) at 2 g/L of RO water buffered on a gram per gram basis with sodium bicarbonate. When a surgical level of anesthesia was achieved, the animal was then briefly dipped in anesthetic-free water and placed in sternal recumbency on a surgical towel moistened with RO water. The area over the right mid-dorsum was cleaned with sterile saline. A 6 mm diameter punch biopsy (Miltex Inc., York, PA, 17402, USA) was utilized to create a full-thickness dermal wound over the right mid-dorsum, sparing the underlying musculature. All biopsies were performed by a single investigator (KEA). Surgical instruments were sterilized between each animal using a cold sterilant (Cidex, Advanced Sterilization Products, Irvine, CA 92618, USA) as outlined by the manufacturer. An immediate post-biopsy photograph was obtained, and the toad was then transferred to an individual recovery tub containing anesthetic-free RO water. Heart rate via noninvasive doppler ultrasound and anesthetic depth were monitored during anesthesia and recovery.

Following recovery, 33 toads were assigned via lottery to one of three groups: low-level laser therapy (LT), topical silver sulfadiazine (SD), and sham treatment control (CT). For the remainder of the study, toads were housed in three tubs according to their treatment group. This was done to prevent contact of the LT and CT groups with the topical SSD, either directly from the SSD treated toads or via environmental contamination. All three groups remained under identical husbandry protocols. Each toad received meloxicam (Loxicom 5 mg/mL, Norbrook Inc., Overland Park, KS 66210, USA), 0.3 mg/kg IM in a forelimb while under anesthesia and then daily for three days postoperatively.

To provide baseline histologic analysis, a single toad was euthanized immediately following biopsy, prior to anesthetic recovery. Euthanasia was performed by inducing a deep plane of surgical anesthesia via partial submersion in buffered MS-222 at 4.2 g/L followed by intracardiac injection of sodium pentobarbital (Euthasol 390 mg/mL, Virbac Animal Health, Fort Worth, TX 76137, USA) 0.5 mL/kg. Death was confirmed via cessation of cardiac flow as determined by a Doppler ultrasound probe. Tissues were collected and preserved as outlined below.

### 2.3. Treatment Schedule

Treatments were first administered on day 1 after biopsy and then every 72 hours thereafter for a total of nine treatments over 28 days.

### 2.4. Laser

To standardize the laser-to-wound distance and positioning, toads from the LT group were individually placed in an opaque circular polypropylene container and covered with a modified lid containing an approximately 6 cm diameter opening centered over the right mid-dorsum, through which the laser treatment was applied. The toads remained awake for this procedure. A class 3B solid-state super pulsed laser (MR4 ACTIVet veterinary laser, Multi Radiance Medical, Solon, OH 44139, USA) fitted with a dome probe was placed in a hands-free flexible arm and centered perpendicular to the wound at a distance of approximately 1 cm. The laser unit was set to 5 Hz and applied for two minutes for a total dose of 15 joules (delivered by one 905 nm wavelength on a super pulsed sequence, four 875 nm wavelength, and two 660 nm wavelength red LEDs) and a static magnetic field of 35 microtesla. The toads were observed throughout LLLT application. Protective eyewear was worn by personnel administering the LLLT. The modified lid decreased direct ophthalmic exposure to the toads during treatment. Each toad was returned to its enclosure immediately following treatment.

### 2.5. SSD

To standardize handling procedures among all three groups, toads from the SD group were individually positioned in the polypropylene containers as outlined above for the LT group. A thin layer of silver sulfadiazine (SSD 1% cream, Ascend Laboratories, LLC., Parsippany, NJ 07054, USA) was applied directly to the wound using a cotton-tipped applicator. The toad remained in the container for two minutes prior to returning to its enclosure.

### 2.6. Control

Toads in the CT group were individually positioned in the polypropylene containers as outlined for the LT group. Sham laser treatment was applied by positioning the laser unit approximately 1 cm from the wound. Following a period of two minutes, the toad was returned to its enclosure.

### 2.7. Visual Assessment of Wounds

Digital photographs (300 dpi) of each wound were obtained at the time of surgery, day 4 after biopsy, and then every 72 hrs thereafter in conjunction with scheduled treatments (days 7, 10, 13, 16, 19, 22, 25, and 28). Photographs were obtained prior to each treatment session. All photographs were captured at a standard distance (30 cm) perpendicular to the animal. Images were evaluated subjectively based on the presence and character of discharge, visible reepithelialization, gross wound contracture (puckering of skin surrounding the wound), and periwound appearance. All wound evaluation was performed by a single investigator (KEA) blinded to the treatment groups.

### 2.8. Euthanasia and Sample Collection

Toads were euthanized at one of five timepoints to evaluate the microscopic effects of treatment. The timepoints (days 4, 7, 13, 19, and 28 after biopsy) were chosen to best reflect the approximate stages of wound healing (inflammatory, proliferative, and maturation). Two toads from each group were chosen randomly via lottery and euthanized at days 4, 7, 13, and 19 after biopsy. The remaining three toads from each group were euthanized at day 28 after biopsy.

Euthanasia was performed by inducing a deep plane of surgical anesthesia via partial submersion in MS-222 at 3.3 g/L buffered on a gram per gram basis with sodium bicarbonate followed by intracardiac injection of sodium pentobarbital 0.05–0.3 mL (0.3–1.9 mL/kg). Death was confirmed via cessation of cardiac flow as determined by a Doppler probe. The skin and associated subcutaneous tissues encompassing the wound area were sharply dissected and fixed whole in neutral buffered 10% formalin. A gross postmortem examination was performed.

### 2.9. Histology

Fixed skin sections were processed routinely for histology and slides were stained with hematoxylin and eosin. Histologic examination was performed by a pathologist (BT) blinded to the treatment groups. Histologic grading of the wounds was performed on a scale of 0–4 for each of five characteristics: lymphocytic inflammation, granulomatous inflammation, heterophilic inflammation, granulation tissue, and fibrosis. Epithelial thickness and degree of epithelial union (complete or incomplete) were also reported. Histologic grading criteria are presented in Table 1.

### 2.10. Statistics

Statistical analyses were performed using R, version 3.5 (R Foundation for Statistical Computing, 1020 Vienna, Austria) and JMP Pro, version 13.0 software (SAS Institute Inc., Cary, NC 27513, USA). Due to the small sample size, all data were analyzed by nonparametric methods. Paired data (body weight at the beginning and end of the study) were compared using the Wilcoxon signed rank test for matched pairs. Weights among the three groups at the beginning of the study and the day of euthanasia were compared using the Kruskal–Wallis test. A *P* value of 0.05 was used to determine statistical significance.

To increase the statistical power and to reflect three general stages of healing, the histologic scores from the first two and second two timepoints were averaged (days 4 and 7; days 13 and 19). Two-way comparisons of histologic scores between the control and treatment groups at each of the three timepoints were performed using Wilcoxon rank sums (comparing CT to LT and CT to SD). A corrected critical *P* value for multiple comparisons of histologic grading was calculated using a Bonferroni adjustment (*P*=0.01). Statistics were not performed on the subjective visual wound assessments.

## 3. Results

### 3.1. Animals

All animals appeared outwardly healthy throughout the study period. Mean weight was 179 g (range 90–460 g) at the initiation of the study. Body weight did not differ significantly between or within the three groups from the initiation of the study to the time of euthanasia. Frequently, toads exhibited behavioral inflation of lungs or urination upon initial restraint, both of which are common responses to handling in this species. The toads otherwise tolerated treatment and handling. The animals were amenable to temporary restraint during treatment applications and exhibited few movements. Rarely was repositioning of the laser necessary, as toads typically remained still for the two-minute treatment period. There was no evidence of discomfort during laser therapy.

On day 1, a single animal in the control group had a focal area of lung exposed at the cranial margin of the biopsy wound that was visible only during inspiration, indicating coelomic exposure. Coelomic exposure was not visible immediately postoperatively, suggesting that additional conspecific trauma or self-trauma may have contributed to coelomic breach. The coelomic exposure was not appreciable on day 4, and by day 7 there was visible epithelium covering the entire wound bed. This animal exhibited no changes in demeanor, weight, or appetite during the study, and the wound appeared to heal appropriately until euthanasia on day 28. Therefore, it was included in statistical analyses.

### 3.2. Visual Assessment of Wounds

On day 4, ten of 33 toads had a thin rim of new epithelium visible at the margin of the wound bed (6/11 LT, 2/11 SD, and 2/11 CT), and all wounds appeared moist with scant, translucent discharge. On day 7, 25/27 toads had visible epithelium covering 50% or more of the wound bed (9/9 in LT, 7/9 in SD, and 9/9 CT). On day 10, 21/21 toads had visible epithelium covering 75% or more of the wound bed. On day 13 and thereafter, 21/21 toads had visible epithelium covering 100% of the wound bed that persisted until euthanasia. Evidence of wound contraction was first visible on day 13 in 20/21 toads (9/9 LT, 9/9 SD, and 8/9 CT), and visible in all toads by day 16. All wounds appeared to heal over the course of the study. Changes to the periwound area and discharge were not observed.

### 3.3. Histology

**Figure 1:**
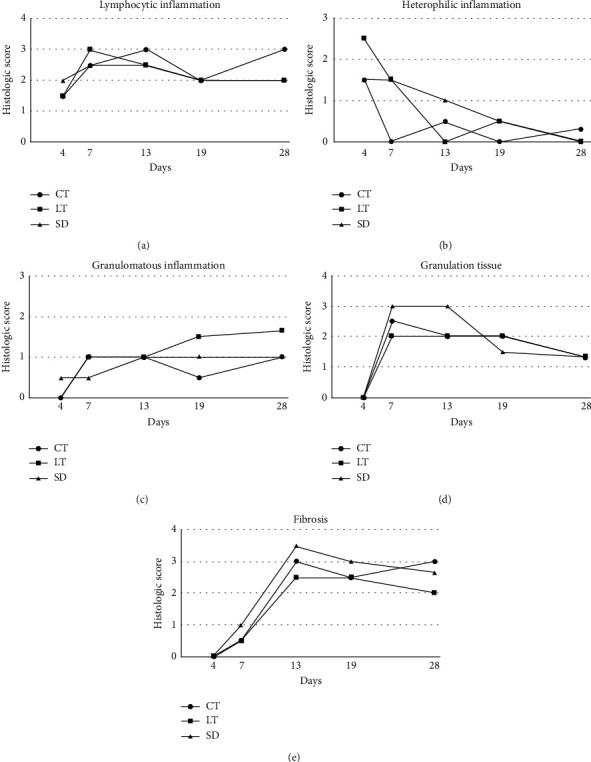
Histologic scores for inflammation, granulation tissue, and fibrosis over time in surgically induced 6 mm diameter full-thickness dermal wounds in marine toads (*Rhinella marina*) treated with low-level laser therapy (LT), topical silver sulfadiazine (SD), or untreated controls (CT). Scores represent an average of two toads from each treatment group for days 4–19. Scores represent an average of three toads from each treatment group for day 28.

**Figure 2:**
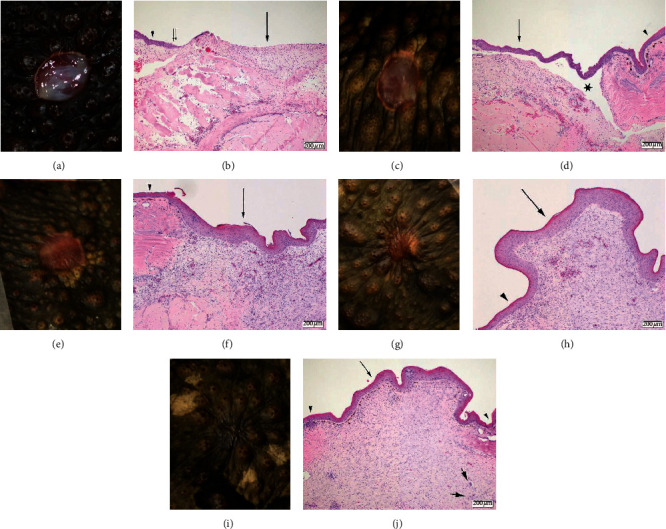
Gross and corresponding histologic images showing the healing progression of surgically induced 6 mm diameter fill-thickness dermal wounds in marine toads (*Rhinella marina*) in the control group at day 4 (a, b), day 7 (c, d), day 13 (e, f), day 19 (g, h), and day 28 (i, j). Histologic sections were stained with hematoxylin and eosin. The scale bar represents 200 micrometers. 2b: there is a thin layer of new epithelial growth (thin double arrows) that extends from the edge of the ulcer (arrowhead). There is a mild, predominately lymphocytic infiltrate, scattered within the tissue below the area of ulceration (arrow). 2d: the area of ulceration is completely covered by new epithelial growth (arrow) which is disorganized and easily separated from the underlying dermis (star) compared to the adjacent skin (arrowhead). A mild lymphocytic infiltrate and small amount of granulation tissue are present within the underlying dermis. 2f: the epithelium covering the previous area of ulceration (arrow) is disorganized and thickened (up to four times the adjacent epidermis (arrowhead)). Granulation tissue and early fibrosis are evident within the underlying dermis which also contains a moderate, diffuse, lymphocytic infiltrate. 2h: similar to 13 days, the new epithelium (arrow) is thicker and disorganized compared to the adjacent dermis (arrowhead). There is moderate fibrosis within the underlying dermis with prominent contracture of the wound resulting in projection of the affected region. 2j: the new epithelium is returning to normal thickness and organization (arrow) relative to the adjacent skin (arrowhead) and the dermal contraction is markedly reduced compared to day 19. Lymphocytes still diffusely infiltrate the dermis and form aggregates along the margin along with rare multinucleated giant cells (thick short arrows). Melanocytes are present within the superficial dermis of the previously ulcerated area; however, no adnexal structures are observed.

There were no statistically significant differences in histologic scores between the control and treatment groups at any of the three points analyzed (days 4 and 7, days 13 and 19, and day 28). The histologic characteristics of all three groups followed general trends expected for wound healing (Figures 1 and 2). Evidence of reepithelialization was present by day 4 in most toads euthanized at that timepoint (2/2 LT, 1/2 SD, and 1/2 CT), with hyperplasia at the margin of the lesion thinning toward the center of the lesion as the new epithelium became more attenuated. By day 7, wounds were completely covered by minimally hyperplastic, disorganized epithelium loosely attached to the underlying dermis resulting in artifactual separation of the dermis and epidermis during tissue processing. Consistent with visual assessments, the epidermis continued to thicken (up to 15 cells thick) through day 28 and remained disorganized with frequent individual cell apoptosis. Granulations tissue within the superficial dermis was absent at day 4, peaking at days 7 and 13, and then slowly decreasing at later timepoints. Dermal fibrosis was evident by day 7 and increased markedly by day 13 along with visible wound contracture; in some toads, the fibrosis was extensive extending up to the underlying bone. The new epithelia lack adnexal structure and a visible Eberth–Katschenko layer.

The initial inflammatory response at day 4 was characterized by mild-to-moderate heterophilic infiltrates. After day 4 through the end of the study, inflammation consisted of a predominately, mild-to-moderate, lymphocytic infiltrate with minimal-to-mild, multifocal aggregates of multinucleated giant cells along the edges of the lesions. Some of the multinucleated cells at the margin of the lesion surrounded visible remnants of the Eberth–Katschenko layer which had become displaced deeper into the dermis. There was a trend of lower lymphocytic inflammation in SD and LT as compared to CT at day 28 (*P*=0.046), but this was not statistically significant. Superficial bacteria were only observed in a single animal (SD group, day 4), and secondary infections were not identified in any sample. Heterophilic inflammation was most severe at day 4 then decreased over the course of healing. Granulomatous inflammation was first identified at day 4 or 7 and remained relatively stable or increased through the remainder of the study. Lymphocytic inflammation was present at day 4 and was increased in all groups at day 7 or 13. Granulation tissue was increased in all groups through day 13. Evidence of fibrosis was absent until day 7 and then generally increased through the healing period.

## 4. Discussion

LLLT has recently gained popularity in veterinary medicine as an adjunct treatment for a variety of conditions including postoperative pain, soft tissue injury, and dermal wounds. While multiple controlled investigations have found that LLLT can improve healing time in mammals [28–34], a recent study in domestic dogs found no apparent improvement in acute healing of wounds treated with this modality [36]. Furthermore, very few studies have evaluated the utility of LLLT in nonmammalian species. Reports of LLLT in reptiles have found limited benefits when applied to dermal wounds [37–39]. Specifically, in a 30-day study examining primary closure of surgically induced dermal wounds in ball pythons (*Python regius*), the authors noted improved collagen maturation on day 14, but no additional beneficial effects were identified [38]. Similarly, an investigation into the effects of LLLT or SSD on second-intention healing in green iguanas (*Iguana iguana*) found that wounds treated with LLLT were significantly smaller than those treated with SSD at the end of the 14-day study, but histologically there were no significant differences among treatments [39]. Both species tolerated treatment application without evidence of negative effects, suggesting that alternative dosing strategies could be pursued to fully evaluate the use of LLLT in reptiles.

The authors are not aware of any previous studies evaluating the safety and efficacy of LLLT in amphibians. Amphibians share some basic epithelial traits with mammals and reptiles, but their relatively thin skin and lack of protective dermal structures may increase their risk of injury from laser therapy [40]. The laser settings utilized in the current study were based on the manufacturer's recommendations. The laser was held approximately 1 cm from the wound bed to reduce the power at the treatment area and therefore decrease the risk of side effects. The 72 hr dosing schedule was chosen based on what would be considered practical for long-term (4 wk) wound management in a clinical case. The dose and application method used did not cause adverse effects based on gross and histologic examination, and the study animals appeared tolerable of treatment application.

In contrast to the relatively new LLLT, SSD is a common topical antimicrobial used in amphibians under human care. In human medicine, SSD was historically considered a first-line therapy for burn wounds, although current literature and meta-analyses have found that SSD can impair wound healing, namely, by inhibiting reepithelialization and via cytotoxic effects [41–44]. Similarly, recent investigations in rats and rabbits found that SSD retards reepithelialization and slowed healing in surgically induced wounds [45, 46]. Therefore, SSD may not be indicated for the inflammatory and/or proliferative phases in noninfected wounds, and its benefit as an antimicrobial agent must be weighed against the potential impairment of wound healing. Amphibians exhibit healing phases comparable to those of mammals, including the inflammatory phase, proliferative phase, and maturation phase [10]. In the investigators' experiences, SSD is often applied to amphibian patients throughout the healing process to treat and prevent infection, though the effect of SSD on granulation tissue formation and epithelialization in amphibians is unknown.

In the current study, gross and histologic examination of surgically induced wounds in marine toads revealed clinically acceptable healing progression in all three treatment groups (LT, SD, and CT), with no significant differences in histologic scores between the treatment and control groups at any timepoint. Epithelium was visible at the margin of the wound as early as day 4 after biopsy. Subjective wound assessment found that more toads in the laser treatment group had epithelium present at the wound margin on day 4 compared to SD and CT toads. This was consistent with histologic findings of the six toads euthanized on day 4. This suggests that LT may improve the rate of reepithelialization in this species, though statistics were not performed on this single timepoint due to the small sample size. By day 13, all wound beds had completely reepithelialized based on visual assessment. This finding correlated with microscopic presence of reepithelialization in toads euthanized on day 13 and later.

Despite the risk of environmental pathogen exposure, secondary bacterial or fungal infection was not observed, and only one animal showed superficial bacterial colonization. The basic pattern of healing and inflammation followed those previously reported for *Xenopus laevis* [10]. Relative to the adjacent tissue, the new epithelium was hyperplastic and lacked adnexal structures and an Eberth–Katschenko layer. The Eberth–Katschenko layer is a calcified dermal structure found in many anurans, the function of which is not yet known. Interestingly, small remnants of the Eberth–Katschenko layer within the disrupted tissue elicited a granulomatous response. Inflammation and fibrosis progressed similarly in all groups. Therefore, LLLT and SSD did not appear to provide a benefit in terms of gross and histologic evidence of wound healing as compared to no treatment. These findings may not justify the financial cost of LLLT treatment or the potential stress of repeated restraint for topical SSD treatment applications when used on uncomplicated skin wounds in marine toads. Further, epithelium serves as a natural barrier to pathogens, so the application of SSD onto the fragile, newly formed epithelial cells may cause unnecessary disruption to this barrier.

Several limitations were encountered in this pilot study, namely, the small sample size. Allowing individual toads to act as their own controls by creating 2-3 wounds per animal was considered for this study. This is an attractive model and is commonly utilized in wound healing studies [36, 38]; however, LLLT is reported to induce systemic effects that may improve the healing of distant wounds [47], thereby precluding the use of multiple treatments per animal. It is possible that the creation of a larger wound bed may have allowed for discrimination among treatment effects, specifically the rate of epithelialization. Further, the lack of significant findings in the LT and SSD groups may simply be a result of this species' robust healing capabilities. In particular, SSD is used for the treatment and prevention of local infection; therefore, uncomplicated wounds may not benefit from this therapy after reepithelialization. Naturally occurring or infected wounds may serve as better models for the effects of SSD on healing characteristics in amphibians. Additionally, it is possible that the stress of handling mitigated any potential beneficial effects of the treatments. Future investigations in similar species may benefit from a control group that is not subjected to routine handling. Alternatively, the design of the treatment modalities may have represented inappropriate or inadequate dosage/frequency of treatment to resolve statistical significance.

## 5. Conclusion

Comparable to recent findings in reptiles, the application of LLLT and SSD appeared to be safe and well-tolerated in marine toads, but was not found to significantly improve the healing characteristics of surgically induced full-thickness dermal wounds as compared to controls. Therefore, the use of SSD or LLLT may not be warranted for the treatment of uncomplicated dermal wounds in this species. Future studies will benefit from a larger sample size and by examining alternative dosing strategies.

## Figures and Tables

**Table 1:** System for histologic grading of dermal wounds in marine toads (*Rhinella marina*).

Score	Inflammation and granulation tissue	Fibrosis
0	None	None
1	Minimal	Scant bands of collagen
2	Mild	Discrete regions of fibrosis
3	Moderate	Fibrosis extends to the level of bone
4	Severe	Fibrosis extends nearly to peritoneum or into bone

## Data Availability

The data are available upon request to the corresponding author.
